# Increased platelet reactivity in patients with late-stage metastatic cancer

**DOI:** 10.1002/cam4.86

**Published:** 2013-05-21

**Authors:** Niamh M Cooke, Karl Egan, Siobhan McFadden, Liam Grogan, Oscar S Breathnach, John O'Leary, Bryan T Hennessy, Dermot Kenny

**Affiliations:** 1 Molecular and Cellular Therapeutics, The Royal College of Surgeons in IrelandDublin, Ireland; 2 The Biomedical Diagnostics Institute, Dublin City UniversityDublin, Ireland; 3 Department of Medical Oncology, Beaumont HospitalDublin, Ireland; 4 Department of Histopathology, Trinity College DublinDublin, Ireland

**Keywords:** Activation, aggregation, hyperreactivity, metastasis, platelets

## Abstract

Platelet hyperreactivity is associated with an increased risk of thrombosis. Cancer patients are at an increased risk of thrombosis, a risk that increases with disease progression. While cancer patients show evidence of platelet activation *in vivo*, few studies have extensively assessed whether these patients display platelet hyperreactivity. We hypothesized that patients with metastatic cancer would display platelet hyperreactivity, reflecting their associated high risk of thrombosis. In a cohort of patients with metastatic cancer (*n *=* *13), we assessed platelet function using well-established assays of platelet reactivity (agonist-induced platelet aggregation, spontaneous platelet aggregation, and agonist-induced P-selectin expression). In comparison with healthy controls (*n *=* *10), patients with metastatic cancer displayed global platelet hyperreactivity. Agonist-induced platelet aggregation responses to ADP (adenosine diphosphate), epinephrine, collagen, arachidonic acid, and PAR-1 (protease-activated receptor-1) activating peptide, as well as spontaneous platelet aggregation, were significantly increased in patients with metastatic cancer. Furthermore, agonist-induced platelet P-selectin expression was also significantly increased within the patient cohort. We demonstrate that patients with metastatic cancer are characterized by global platelet hyperreactivity, a factor that may contribute to their increased risk of thrombosis.

We assessed platelet function in a cohort of patients with metastatic cancer (*n* = 13) using well-established assays of platelet reactivity. Agonist-induced platelet aggregation and activation in response to platelet agonists, as well as spontaneous platelet aggregation, was significantly increased in cancer patients compared with healthy controls. We demonstrate that patients with metastatic cancer are characterized by global platelet hyperreactivity, a factor that may contribute to their increased risk of thrombosis.

## Introduction

To date, there is no direct evidence of hyperreactive or “primed” platelets in cancer patients. The recently described phenomenon of platelet hyperreactivity can be defined as an exaggerated response to stimuli, that is, increased platelet activation or aggregation in response to low doses of agonists. Increased platelet reactivity has previously been associated with an increased incidence of both venous and arterial thrombosis 1,2. Venous thromboembolism (VTE) is a frequent complication of cancer, and is the second leading cause of death in cancer patients after metastasis 4. Despite the long-established association between VTE and cancer 5–6, there is a lack of platelet function studies in the literature to elucidate the pathophysiologic mechanism behind this relationship. Elevated plasma levels of soluble P-selectin, a marker of platelet activation, have recently been shown to be predictive of VTE in breast, lung, gastrointestinal tract, prostate, and other cancers 7. Other markers of *in vivo* platelet activation, including platelet factor 4 and beta-thromboglobulin, have also been shown to increase with malignancy 8,9. While several studies have shown that cancer patients have increased levels of platelet activation markers, there is limited data available on whether their platelets are more responsive to stimuli.

In this study we assessed platelet reactivity in a cohort of patients with late-stage metastatic cancer. We employed two well-established platelet function assays using incremental concentrations of multiple agonists to determine the propensity of platelets to react to stimuli via different activation pathways, simultaneously. Hence, we could also establish if platelet hyperreactivity was unique to a specific platelet signaling pathway or global. Platelet aggregation was measured using a 96-well plate modification in classical light transmission aggregometry developed in our laboratory 11. To determine if an altered platelet aggregation profile was accompanied by an altered platelet activation profile, we analyzed P-selectin expression in response to agonist stimulation using a modified flow cytometry-based assay 12. We demonstrate that patients with metastatic cancer are predisposed to high levels of platelet reactivity in response to low-dose agonist stimulation. This cohort of patients show direct evidence of global platelet hyperreactivity, which may provide a mechanism for the high level of adverse cardiovascular events observed in cancer patients.

## MATERIALS AND METHODS

### Study design

This study was approved by the Royal College of Surgeons in Ireland and Beaumont Hospital ethics committees. Written, informed consent was obtained from all participants prior to phlebotomy. As thrombosis is associated with a wide range of cancer types 7, we choose a heterogeneous cohort of patients, all of whom had disseminated malignancy, to critically examine the effect of metastasis on platelet function. In total, we studied 10 different cancer types, including sex-specific cancers (e.g., metastatic ovarian cancer). All patients were ≥18 years, with histologically proven incurable stage VI cancer and who had at least one palliative chemotherapy regimen for incurable cancer. The characteristics of the patient group are outlined in Table 1. Ten healthy donors who had not taken medications known to affect platelet function for ≥10 days were recruited. The average healthy donor was 48 years, 70% were >50 years, and 60% were female.

**Table:** Patient characteristics

Age (years)	64 ± 4
Male	8 (62%)
Platelet (×10^6^/mL)	328 ± 34
White blood cell (×10^6^/mL)	8 ± 1
C-reactive protein (mg/L)	27 ± 7
Diagnosis	
Metastatic ovarian cancer, stage IV	2 (15%)
Metastatic pancreatic cancer, stage IV	2 (15%)
Metastatic breast cancer, stage IV	2 (15%)
Metastatic lung cancer, stage IV	1 (8%)
Metastatic colorectal cancer, stage IV	1 (8%)
Metastatic melanoma, stage IV	1 (8%)
Metastatic chondrosarcoma, stage IV	1 (8%)
Advanced gastric cancer, stage IV	1 (8%)
Recurrent esophageal cancer, stage IV	1 (8%)
Glioblastoma multiforme, stage IV	1 (8%)
Medications	
Aspirin	2 (15%)
Anticoagulant	5 (39%)
Paracetamol	4 (31%)
Beta-blocker	3 (23%)
Calcium channel blocker	2 (15%)
Oral nitrate	1 (8%)
Angiotensin-converting enzyme inhibitor	1 (8%)
Cardiac glycoside	1 (8%)
Angiotensin II receptor antagonist	1 (8%)
Statin	2 (15%)
Loop diuretic	2 (15%)
Opiate	8 (62%)
Glucocorticosteroid	4 (31%)
Proton pump inhibitor	8 (62%)
Selective serotonin reuptake inhibitor	3 (23%)
Antibiotic	4 (31%)

Data are presented as mean value ± SEM or number (%) of patients. Information on C-reactive protein was available for 10 patients.

### Platelet preparation

Blood was collected by venipuncture through a 19-gauge butterfly needle without a tourniquet to avoid platelet activation. Platelet-rich plasma (PRP) was obtained from 3.2% trisodium citrated blood (10% vol/vol) centrifuged at 170 *g* for 10 min. Platelet-poor plasma (PPP) was prepared by centrifuging the remaining whole blood at 1500 *g* for 10 min.

### Platelet aggregometry

Platelet aggregation in response to incremental concentrations of five agonists was measured using a 96-well plate modification in classical light transmission aggregometry, as previously described 11. This assay has a major advantage over standard platelet function tests. Platelet aggregation has two phases, a primary response to the agonist added, followed by a secondary response to the secreted agonists from platelet granules. Standard platelet function tests typically include a single agonist, at a maximal concentration, which produces a very intense primary response, thus masking differences in platelet responses per donor.

The agonists used were ADP (adenosine diphosphate), arachidonic acid, soluble calf skin collagen (Type 1), epinephrine (Bio/Data Corporation, Horsham, PA), and TRAP (thrombin receptor-activating peptide: SFLLRNPNDKYEPF-amide, PAR-1 [protease-activated receptor] agonist; Sigma-aldrich, St. Louis, MO). PRP was incubated with increasing concentrations of agonists diluted with JNL buffer (130 mmol/L NaCl, 10 mmol/L sodium citrate, 9 mmol/L NaHCO_3_, 6 mmol/L D-glucose, 0.9 mmol/L MgCl_2_, 0.81 mmol/L KH_2_PO_4_, and 10 mmol/L Tris, pH 7.4). The final agonist concentrations were as follows: ADP: 20, 10, 5, 2.5, 1.25, 0.6, 0.3, and 0.15 μmol/L; arachidonic acid: 1.64, 1.23, 0.62, 0.31, 0.15, 0.08, 0.04, and 0.02 mmol/L; collagen: 190, 142.5, 71.25, 35.6, 17.8, 8.9, 4.4, and 2.2 μg/mL; epinephrine: 20, 5, 1.25, 0.3, 0.07, 0.01, 0.004, and 0.001 μmol/L; and TRAP: 20, 10, 5, 2.5, 1.25, 0.6, 0.3, and 0.15 μmol/L. PRP and PPP were included as positive and negative controls, respectively.

The plate was incubated at 37°C and shaken vigorously. The optical density (OD) of each well was measured at 572 nm using a Victor 2 V Multilabel Counter plate reader (PerkinElmer, Waltham, MA) at 0, 3, 9, 15, and 18 min. The absorbance or OD values were subsequently transformed into percentage platelet aggregation values, using the light absorbance of PPP and PRP as reference for 100% and 0% aggregation, respectively. Results are expressed as the maximum aggregation response achieved over the five time points. The concentration of agonist which yields a response halfway between baseline and maximum aggregation (EC50) was calculated to determine responsiveness. Spontaneous platelet aggregation (SPA) was also measured using this assay. SPA was measured in the PRP control wells over the assay time course in the absence of agonist stimulation, but in the presence of mechanical stimulation, that is, shaking in the plate reader. A decrease in light absorbance in these PRP wells from time 0 to 18 min was considered to indicate positive SPA.

### Platelet activation assay

Agonist-induced platelet activation, as determined by the percentage of P-selectin positive platelets, was assessed using a flow cytometry–based assay modified from that described by Nylander et al. 12. Whole blood was diluted 1:10 with JNL buffer, supplemented with 1.8 mmol/L CaCl_2_. It was incubated with either no agonist, ADP (1 and 5 μmol/L), ADP/epinephrine (both at 1 and 5 μmol/L), or TRAP (1, 2, and 20 μmol/L), in the presence of 1.25 μg/mL PE (phycoerythrin)-labeled antihuman P-selectin antibody or an appropriate isotype control (BD Biosciences, San Diego, CA). All incubations were performed at room temperature for 10 min. The reaction was terminated with 1 mL of JNL buffer. Samples were analyzed within 1 h using a BD FACS Calibur (Becton Dickinson). Data were analyzed using CellQuest Pro software and activation of platelets was expressed as the percentage of platelets that were P-selectin positive relative to the isotype control.

## Results

### Platelet aggregation is significantly increased in patients with metastatic cancer compared with healthy donors

Agonist-induced platelet aggregation in patients with metastatic cancer and healthy controls was measured using a 96-well platelet modification in classical light transmission aggregometry. This PRP-based assay determines the propensity of platelets to aggregate in response to multiple concentrations of five agonists, and hence tests multiple platelet activation pathways simultaneously.

**Figure 1:**
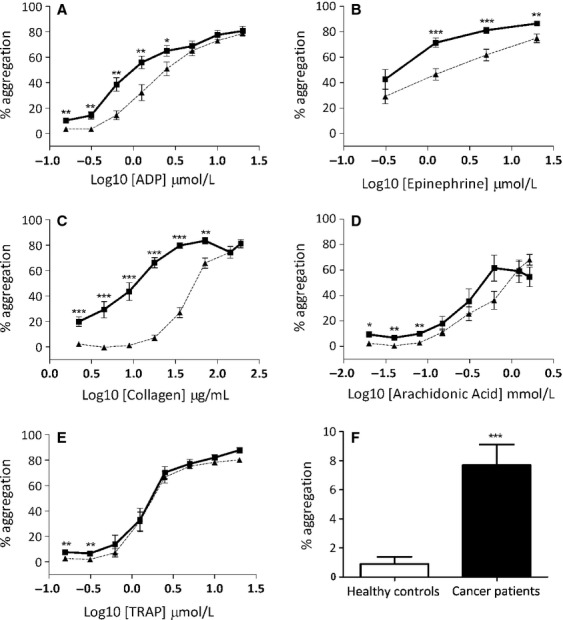
Platelet aggregation is significantly increased in patients with metastatic cancer compared with healthy donors. (A–E) Platelet aggregation responses (*y*-axis) to increasing concentrations of ADP, epinephrine, collagen, arachidonic acid, and TRAP (*x*-axis), respectively, were measured in patients with metastatic cancer (squares with solid line, *n* = 13) compared with healthy donors (triangles with broken line, *n* = 10). (F) Spontaneous platelet aggregation response (*y*-axis) in the absence of agonist stimulation was measured in patients with metastatic cancer (solid bars, *n* = 13) compared with healthy donors (open bars, *n* = 10) (*x*-axis). Data shown are mean ± SEM. *P ≤* *0.05, **P ≤* *0.01, and ***P ≤* *0.001 were determined by Student *t* test.

For each agonist there was a dose-dependent increase in platelet aggregation for both cohorts (Fig. 1A–E). Overall, patients with metastatic cancer displayed significantly increased platelet aggregation responses to ADP, collagen, arachidonic acid, TRAP, and epinephrine compared with healthy controls. This was predominantly evident at low- and mid-range agonist concentrations. In response to ADP stimulation, cancer patients displayed significantly increased platelet aggregations at concentrations between 0.15 and 2.5 μmol/L (Fig. 1A). Similarly, the greatest differences in platelet aggregation were observed at low concentrations of collagen (2.2–71.25 μg/mL, Fig. 1C), arachidonic acid (0.02–0.08 mmol/L, Fig. 1D), and TRAP (0.15–0.3 μmol/L, Fig. 1E). Platelet aggregation responses to high concentrations of these four agonists yielded no difference between the two cohorts.

The high level of platelet aggregation observed in patients with metastatic cancer is reflected by the EC50 value of each agonist, that is, the concentration of agonist which yields a response halfway between baseline and maximum aggregation. The EC50 value of ADP was 0.6 μmol/L in cancer patients compared to 1.7 μmol/L in healthy controls. This trend was also observed for the EC50 value of collagen (5.6 μg/mL vs. 84.3 μg/mL) and arachidonic acid (0.3 mmol/L vs. 1.3 mmol/L). There was no difference in the EC50 value of TRAP between the two cohorts (1.5 μmol/L vs. 1.4 μmol/L). Therefore, significantly lower concentrations of ADP, collagen, and arachidonic acid are needed to produce an exaggerated aggregation response in the patient cohort. In contrast to other agonists, epinephrine-induced platelet aggregation was increased in cancer patients in response to high concentrations, as well as low-range concentrations (Fig. 1B). The EC50 value of epinephrine was also considerably lower in patients with metastatic cancer compared with healthy controls (0.1 μmol/L vs. 1.7 μmol/L).

### SPA is significantly increased in patients with metastatic cancer compared with healthy donors

An increase in SPA is indicative of increased platelet reactivity. It has previously been associated with an increased incidence of thrombosis and poor prognosis in patients who have survived a recent myocardial infarction 2. SPA in patients with metastatic cancer and healthy controls was also measured using the 96-well plate modification in light transmission aggregometry. SPA was significantly increased within the cancer patient cohort (*P *≤* *0.001; Fig. 1F).

### Platelet activation is significantly increased in patients with metastatic cancer compared with healthy donors

To establish if increased platelet reactivity was evident across different platelet functions assays, which employ different experimental parameters, we also examined levels of platelet activation using whole blood. Agonist-induced platelet activation, as determined by the percentage of P-selectin–positive platelets, was assessed using a flow cytometry-based assay. P-selectin is stored internally in alpha-granules of resting platelets and is translocated to the surface upon activation. This assay determines the propensity of platelets to activate in response to multiple concentrations of three agonists. The concentrations of agonists included were chosen based on preliminary experiments designed to identify concentrations of agonists that induced low (10–30%) and intermediate (30–50%) levels of P-selectin expression in healthy controls.

**Figure 2:**
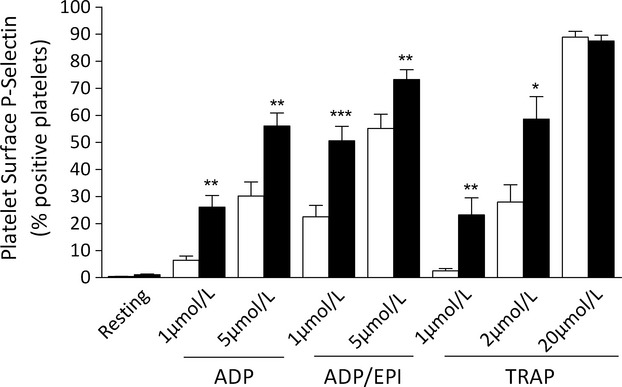
Platelet activation is significantly increased in patients with metastatic cancer compared with healthy donors. Platelet activation in patients with metastatic cancer (solid bars, *n* = 13) compared with healthy donors (open bars, *n* = 10) was determined by platelet surface expression of P-selectin (*y*-axis) in response to single (ADP and TRAP) or combination (ADP/EPI; epinephrine) platelet agonists at multiple concentrations (*x*-axis). Data shown are mean value ± SEM. *P ≤* *0.05, **P ≤* *0.01, and ***P ≤* *0.001 were determined by Student *t* test.

There was no significant difference in basal P-selectin expression between cancer patients and healthy donors (1.1 ± 0.3% vs. 0.4 ± 0.1%; Fig. 2). In response to agonist stimulation, the percentage of activated platelets was significantly increased in cancer patients (Fig. 2). Platelets in patients with metastatic cancer compared with healthy donors were significantly more reactive to low concentrations of ADP (1 μmol/L: *P* ≤ 0.01 and 5 μmol/L: *P* ≤ 0.01), ADP in combination with epinephrine (1 μmol/L: *P* ≤ 0.001 and 5 μmol/L: *P* ≤ 0.01), and also TRAP (1 μmol/L: *P* ≤ 0.01 and 2 μmol/L: *P* ≤ 0.05). There was no significant difference in platelet reactivity to 20 μmol/L TRAP. This concentration of TRAP is known to induce maximal platelet P-selectin expression and thus was included as a positive control.

## Discussion

The results of this study demonstrate for the first time that patients with metastatic cancer display global platelet hyperreactivity. While several studies have shown that cancer patients show increased levels of platelet activation markers, reflecting *in vivo* platelet activation, there is limited data on whether their platelets are more responsive to stimuli. We demonstrate that patients with metastatic cancer have increased agonist-induced platelet aggregation, increased SPA, and increased agonist-induced P-selectin expression. The results are strengthened by the use of different platelet function assays and multiple platelet agonists. A strong correlation between platelet aggregation and activation was observed as these two processes are closely linked, with the initial phase of aggregation involving platelet activation. Addition of an agonist to PRP or whole blood leads to platelet activation, causing platelets to change in their shape from discoid to spiny spheres. Activation is followed by platelet granule release and synthesize of positive feedback mediators, for example, thromboxane A2 and platelet activating factor, which are potent platelet aggregating agonists. This results in activation and recruitment of additional platelets, which coupled with the binding of fibrinogen, mediates aggregation.

We show that patients with metastatic cancer exhibit significantly increased platelet aggregation in response to ADP, epinephrine, collagen, arachidonic acid, and, to a lesser extent, TRAP (Fig. 1A–E). Each agonist stimulates platelets via different platelet surface receptors, which subsequently leads to platelet aggregation via a specific pathway. This suggests that hyperreactivity is a global characteristic of platelets and is not attributed to any one unique agonist/aggregation pathway. Consistent with this finding, our group has previously used this assay to show increased platelet reactivity in response to one or more agonists in patients with HIV, inflammatory arthritis, and cardiovascular disease, which are all cohorts at increased risk of thrombosis 11,13. The most exaggerated increased aggregation profile was seen in response to collagen. Collagen-induced platelet responses are known to be modulated in inflammatory conditions 15. The cancer patients in this cohort displayed significant signs of inflammation (elevated CRP levels, Table 1) that could contribute to changes in platelet reactivity. However, the underlying cellular mechanism of increased platelet aggregation in response to collagen in this cohort is currently unknown and warrants further investigation.

Compared with healthy donors, patients with metastatic cancer also displayed significantly increased SPA (Fig. 1F). SPA is indicative of increased platelet reactivity and is associated with an increased incidence of thrombosis and poor prognosis. Previously, Trip et al. 2 reported that patients with a recent myocardial infarction who were positive for SPA had a significantly higher overall recurrent rate and mortality rate compared with those negative for SPA. They propose that SPA could be a useful biomarker for the prediction of coronary events and mortality in survivors of a myocardial infarction. Our results suggest that a similar observation may be the case for patients with metastatic cancer.

In addition to increased aggregation profiles, we demonstrate that platelets in patients with metastatic cancer are significantly more reactive to ADP, ADP/epinephrine, and TRAP, as determined by P-selectin expression (Fig. 2). A similar observation has only been reported once in the literature. Mantur et al. 16 reported an increase in the expression of P-selectin on platelets in response to ADP and TRAP in patients with renal carcinoma. They also detected significantly higher levels of P-selectin–positive platelets in patients with disseminated malignancy.

In our study, it is not clear why patients with metastatic cancer have significantly higher numbers of activated platelets. The role of platelet turnover in platelet hyperreactivity was not directly assessed. There were no cases of thrombocytopenia or thrombocytosis, all patients had platelet counts within the normal range. Alterations in platelet surface receptors are also unlikely to explain high P-selectin expression as similar results were obtained with all three agonists employed (ADP, epinephrine, and TRAP), each of which activate platelets through a different pathway. It is most likely due to the extracellular platelet microenvironment. Platelets from patients with metastatic cancer may have a “pro-thrombotic” tendency that becomes clinically evident upon agonist stimulation and thus increases the likelihood of VTE. Indeed, Mantur et al. 16 state that increased P-selectin expression in patients with renal cancer is due to the presence of intensified thrombogenesis and other platelet agonists in the blood. Furthermore, the critical contribution of P-selectin to metastasis has previously been shown using mouse models 17–18. A key characteristic of metastatic progression is enhanced expression of tumor cell surface carbohydrates, such as sialyl Lewis. Interaction of P-selectin with these ligands can promote tumor growth, provide protection from immune surveillance, and facilitate proliferation at a secondary site.

Increased agonist-induced platelet activation is also associated with increased thrombosis. Kabbani et al. 1 classified patients undergoing percutaneous coronary intervention as high or low responders to 0.2 μmol/L ADP based on the extent of platelet integrin αIIbβ3 activation. The incidence of a myocardial infarction, urgent revascularization, or repeat revascularization was significantly more prevalent in high responders compared with low responders. Similarly, Furman et al. 19 reported significantly increased platelet reactivity in patients with stable coronary artery disease compared with healthy controls in response to suboptimal concentrations of ADP, ADP/epinephrine, and TRAP.

A number of patients were receiving either antiplatelet therapy (aspirin, *n* = 2) or anticoagulant therapy (*n* = 5). Interestingly, we did not observe a significant decrease in either platelet activation or aggregation in patients receiving these medications compared with healthy controls in response to ADP, epinephrine, collagen, or TRAP. However, there was a decrease in arachidonic acid–induced aggregation in patients receiving aspirin. Similarly, other patients were receiving medications to treat hypertension, angina, high cholesterol, and edema, all of which contribute to alterations in the hemostatic pathway and, therefore, may play a role in impeding platelet function. However, these patients did not demonstrate a decrease in platelet reactivity. Thus, we can conclude that the significant increase in platelet hyperreactivity observed in patients with metastatic cancer was independent of their cardiovascular medications.

Together these data show significant evidence of global platelet hyperreactivity in patients with metastatic cancer. Further studies are warranted to elucidate the molecular mechanism underlying platelet hyperreactivity in these patients. Also, it remains largely unclear if these observations reflect the effect of malignancy on platelet hyperreactivity, or the effect of platelet hyperreactivity on malignancy, or if indeed both. Global platelet hyperactivity could explain the associated high level of adverse thrombotic events in this patient cohort. Clinical trials of currently available antiplatelet agents may represent a novel therapeutic strategy for the treatment of cancer-associated thrombosis.
